# Renal Nerve Stimulation Predicted Blood Pressure–Lowering Responses to Percutaneous Renal Denervation

**DOI:** 10.1161/CIRCINTERVENTIONS.122.012779

**Published:** 2023-02-21

**Authors:** Hui-Chun Huang, Heng-Yu Pan, Tzung-Dau Wang

**Affiliations:** 1 Division of Cardiology, Department of Internal Medicine, National Taiwan University Hospital and National Taiwan University College of Medicine, Taipei (H.-C.H., T.-D.W.).; 2 Graduate Institute of Epidemiology and Preventive Medicine, College of Public Health, National Taiwan University, Taipei (H.-C.H.).; 3 Division of Cardiology, Department of Internal Medicine, National Taiwan University Hospital Hsin-Chu Branch, Hsin-Chu City (H.-Y.P.).; 4 Division of Hospital Medicine, Department of Internal Medicine, National Taiwan University Hospital, Taipei (T.-D.W.).

**Keywords:** ambulatory blood pressure monitoring, clinical application, hypertension, renal denervation, renal nerve stimulation

Renal denervation (RDN) is an alternative treatment strategy for blood pressure (BP) control. The most challenging task about the clinical application of RDN is the identification of reliable response predictors and ablation end points.^1^ We herein evaluated the associations between intra-procedural renal nerve stimulation (RNS)-induced systolic blood pressure (SBP) changes immediately before and after RDN and 24-hour SBP reductions 6 months following RDN.

From February 2019 to January 2021, 16 patients (mean age, 50.5±10.2 years; 10 were men) with uncontrolled hypertension who underwent radiofrequency RDN at the National Taiwan University Hospital were prospectively included. RNS was performed immediately before and after RDN in bilateral proximal (approximately the midpoint of the proximal half of main renal artery) and distal (≈ 1 cm proximal to the bifurcation of branch renal arteries) main renal arteries using the 4 Fr quadripolar catheter (Advanced St. Jude Fix 4 polar Supreme 4F JSN), with bipolar stimulation from poles 1 to 2 (Figure [A]), under general anesthesia by intravenous infusion of a therapeutic dose (0.5–0.7 ml/kg/h) of propofol.^2^ The pacing frequency was set at 10 Hz and the pacing output at 20 mA, with a pulse duration of 10 ms. All patients underwent 24-hour ambulatory BP monitoring at baseline and 6 months after RDN. Antihypertensive medications as prescribed at baseline were required to be taken at least 24 hours before ambulatory BP monitoring performed 6 months after RDN to make the results comparable under the same medication burden. The responder was defined as 24-hour SBP reduction of ≥10 mmHg 6 months following RDN. The primary analysis was to evaluate the associations between RNS-induced SBP changes immediately before and after RDN with 24-hour SBP reductions 6 months following RDN. The study protocol was approved by the Institutional Research Ethics Committee and all patients provided written informed consent. Statistical analyses were performed on SAS software (version 9.4; SAS Institute, Inc, Cary, NC). The data that support the findings of this study are available from the corresponding author upon reasonable request.

**Figure.**
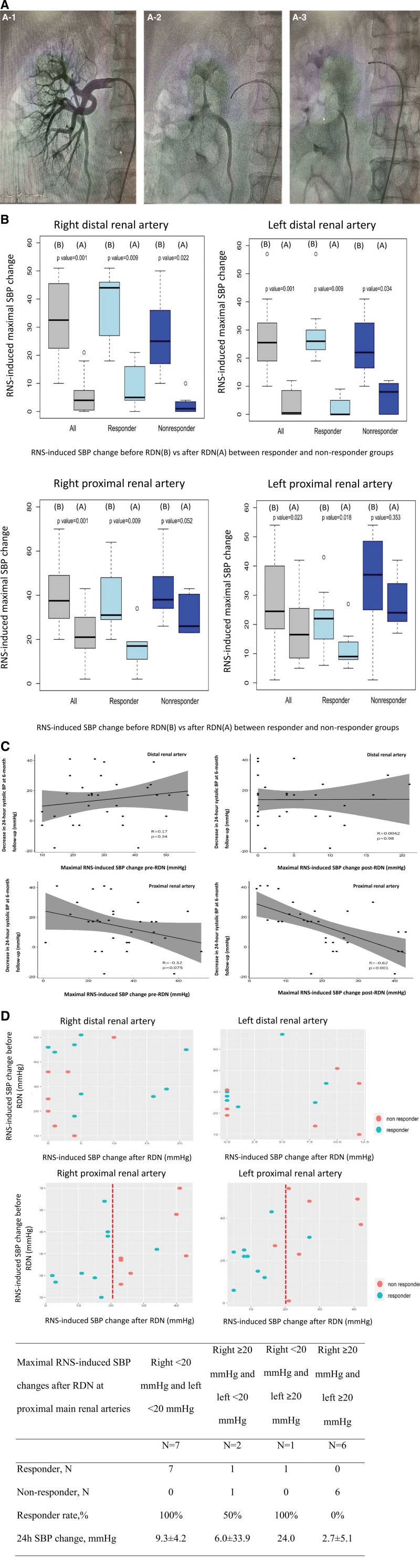
**Renal nerve stimulation (RNS) during renal denervation (RDN) and 6-mo 24-h systolic blood pressure (SBP) changes. A**, Catheter positions and renal angiograms in renal nerve stimulation test. A1: right renal angiogram; A2: right distal main renal artery stimulation site; A3: right proximal main renal artery stimulation site. **B**, Maximal SBP response to RNS before (**B**) and after (**A**) RDN between responder and nonresponder groups among 4 different stimulation sites. **C**, Pearson correlation coefficients for RNS-induced maximal SBP responses and 24-h SBP changes at 6-mo following RDN. **D**, RNS-induced maximal SBP responses before and after RDN in both responder and nonresponder groups. (*Continued* )

Overall, the mean 24-hour SBPs were 144.4±16.1 and 130.6±14.8 mmHg at baseline and 6 months after RDN, respectively (*P*=0.003). Nine (56%) patients were responders. The mean antihypertensive medication burden was 2.2±1.5 at baseline.^3^ Before RDN, an increase in SBP following RNS was universally observed at all stimulation sites and in all patients (Figure [B]). A maximal SBP increase of >20 mmHg in the proximal main renal artery in at least one side was noted in all patients. The maximal SBP increase occurred on average 1.2 minutes after the 1-minute RNS period. Transient BP drop, but soon elevated, was noted in 6 patients. The heart rate changes were non-significant at all sites (all *P*>0.05).

The median number of ablations were 55 (range, 33–90) and 47.5 (range, 28–69) in the right and left renal arteries, respectively. The median ablation number in bilateral branch and distal main renal arteries was 90. After RDN, the rise in RNS-induced SBP was significantly blunted in distal main renal arteries (all *P*<0.05), with 5 cases having RNS-induced SBP increases of >10 mmHg. The RNS-induced SBP changes after RDN over the proximal main renal arteries were less dramatic, albeit statistically significant (Figure [B]). In contrast to the SBP changes in distal main renal arteries, the maximal RNS-induced SBP changes in proximal main renal arteries after RDN were significantly lower in the RDN responder group (right: 15.3±9.6 versus 31.3±9.5 mmHg; *P*=0.005; left: 11.6±6.9 versus 27.6±10.0 mmHg; *P*=0.002; Figure [B]). The sum of maximal RNS-induced SBP increases after RDN in proximal main renal arteries was significantly associated with the 6-month 24-hour SBP change, with a correlation coefficient of −0.62 (*P*<0.001; Figure [C]). Neither the RNS-induced maximal SBP increases before RDN nor the differences in RNS-induced maximal SBP changes before and after RDN correlated with the decrease in 24-hour SBP at 6-month follow-up. The associations between RNS-induced SBP changes and 6-month office SBP changes were consistent with what were observed with 24-hour SBP changes (data not shown). The scatter plot shows the distribution of pre-RDN and post-RDN maximal RNS-induced SBP changes at different sites between responders and non-responders (Figure [D]). All 7 patients with an RNS-induced SBP increase of <20 mmHg in both proximal main renal arteries after RDN were exclusive responders (Figure [D]). The differences of post-RDN RNS-induced maximal SBP changes in bilateral proximal main renal arteries between responders and non-responders remained across different definitions (≥15 mmHg and ≥10%) of responders (data not shown).

Our study shows that RNS-induced SBP increase of <20 mmHg in the proximal main renal artery bilaterally immediately after RDN can predict a good BP-lowering response to RDN and serve as a procedural end point. We designed a randomized clinical trial (URL: https://www.clinicaltrials.gov; NCT 05421767) to compare an RNS-guided RDN strategy, featured by repeat ablations of main renal artery at different sites if the immediate post-RDN RNS-induced SBP response is not blunted to examine whether such an RNS-guided RDN strategy could achieve better clinical outcomes.

## Article Information

### Sources of Funding

Supported by grants from the National Taiwan University Hospital.

### Disclosures

None.
